# Exploring the role of *TWIST1* in malocclusion and craniofacial morphology

**DOI:** 10.3389/fphys.2026.1749243

**Published:** 2026-02-17

**Authors:** Clarissa S. G. Da Fontoura, Steven Eliason, Brad A. Amendt, Aline L. Petrin, Lina M. Moreno Uribe

**Affiliations:** 1 Department of Restorative Sciences, Cariology and Endodontics, School of Dentistry, University of Michigan, Ann Arbor, MI, United States; 2 Department of Anatomy and Cell Biology, Carver College of Medicine, University of Iowa, Iowa City, IA, United States; 3 Iowa Institute for Oral Health Research, College of Dentistry and Dental Clinics, University of Iowa, Iowa City, IA, United States; 4 Department of Orthodontics, College of Dentistry and Dental Clinics, University of Iowa, Iowa City, IA, United States

**Keywords:** genetics, growth and development, morphometrics, orthodontics, polymorphisms

## Abstract

**Objective:**

Despite increasing evidence that common genetic variation contributes to variation in jaw and cranial base morphology, the biological mechanisms underlying malocclusion remain poorly defined. This study tested the hypothesis that a noncoding variant near *TWIST1* alters craniofacial development by disrupting transcriptional regulation, contributing to skeletal phenotypes associated with malocclusion.

**Methods:**

In a cohort of 277 non-syndromic individuals with malocclusion, we performed targeted genotyping and deep sequencing of the *TWIST1* locus, followed by multivariate genotype–phenotype correlation analyses. To evaluate regulatory function, we performed luciferase reporter assays and chromatin immunoprecipitation in multiple cell lines. Craniofacial consequences of *Twist1* loss of function were characterized using 3D morphometrics and craniometric analysis in conditional knockout mice at postnatal days 14 and 21.

**Results:**

The SNP rs2189000, located 4.2 kb upstream of *TWIST1*, showed a significant association with mandibular and anterior cranial base shape (P = 0.0003). No coding mutations were detected. Functional assays revealed that rs2189000 disrupts a conserved *PITX2* binding site, abolishing *PITX2*-mediated activation of *TWIST1* transcription. In mice, mesoderm-specific deletion of *Twist1* produced craniofacial changes, such as domed skulls, mandibular shortening, palatal rotation, and facial asymmetry, that paralleled the human phenotypic associations. Additionally, premature closure of the cranial base synchondroses was observed, indicating a mechanistic link to disrupted postnatal growth trajectories.

**Conclusion:**

This study identifies a putative functional noncoding variant that dysregulates *TWIST1* via disruption of *PITX2* DNA binding and links this effect to postnatal craniofacial phenotypes in both humans and mice. These findings expand the developmental and genetic framework for understanding malocclusion and suggest a broader role for *TWIST1* in cranial base growth and midface patterning.

## 1 Introduction

Malocclusion affects individuals across all racial and ethnic backgrounds and involves disruptions to both hard and soft tissues of the craniofacial complex. Despite increasing recognition of its genetic component, the mechanisms by which common variation influences craniofacial morphology remain poorly defined (Nikopensius, et al., 2013; Guan, et al., 2015; Perillo, et al., 2015; Chen, et al., 2015; Kantaputra, et al., 2019). Unlike rare Mendelian disorders, most skeletal malocclusions arise from polygenic contributions that are incompletely understood.

In our prior study, we conducted a candidate gene analysis of lateral cephalometric radiographs in individuals with malocclusion (da Fontoura, et al., 2015). Among 273 tested variants, we identified a significant association between a noncoding single nucleotide polymorphism (SNP) located 4.2 kb upstream of the *TWIST1* transcription start site (rs2189000 A>G) and a pattern of craniofacial variation characterized by shorter mandibular ramus height, increased mandibular body length, and a steeper anterior cranial base angle (P = 0.000076). Although the phenotypic variation associated with this component explained approximately 8.2% of the total variance in the sample, the biological plausibility of the association was strengthened by overlap with known features of syndromic craniofacial disorders involving *TWIST1*. This finding served as the rationale for the current investigation.

*TWIST1* encodes a highly conserved basic helix-loop-helix (bHLH) transcription factor that plays essential roles in craniofacial development. During murine embryogenesis, *Twist1* is first expressed at E7.5 in the cranial mesoderm and later in neural crest-derived mesenchyme (Stoetzel, et al., 1995). Homozygous *Twist1*-null embryos die mid-gestation due to failure of cranial neural tube fold fusion (Chen and Behringer, 1995). While these embryonic phenotypes are severe, postnatal studies of *Twist1* haploinsufficiency and tissue-specific knockouts have revealed persistent roles in skeletal morphogenesis. In particular, *Twist1* is required for the development and patterning of both the cranial base and mandible, with conditional loss in the cranial mesoderm (CM) or neural crest (NC) lineages leading to cranial asymmetry, mandibular hypoplasia, altered cranial base angle, and abnormal suture and synchondrosis development (Bildsoe, et al., 2013; Funato, 2020; Bourgeois, et al., 1998; Hermann, et al., 2012; Bildsoe, et al., 2009).

*Twist1* also functions as a repressor of premature osteogenic differentiation by antagonizing *Runx2*, a key osteoblast regulator (Bialek, et al., 2004). Through this mechanism, *Twist1* coordinates the timing of intramembranous and endochondral ossification in the cranial vault and base, thereby maintaining suture patency and regulating calvarial bone development (Rice, et al., 2000). In Twist1-haploinsufficient mice, early ossification of the spheno-occipital and intersphenoid synchondroses has been observed, leading to cranial base shortening and facial asymmetry (Behr, et al., 2011; Funato, 2020).

In humans, heterozygous coding mutations in *TWIST1* cause Saethre–Chotzen syndrome (SCS), a craniosynostosis disorder that includes coronal, lambdoid, or metopic suture fusion, maxillary hypoplasia, steep mandibular plane angles, and midfacial asymmetry (Johnson, et al., 1998; Oram and Gridley, 2005; El Ghouzzi, et al., 1999). Cephalometric studies of SCS patients consistently demonstrate reduced ramus height, a steep anterior cranial base, and altered mandibular plane angulation, phenotypes that strongly resemble those observed in individuals carrying the rs2189000 variant in our non-syndromic cohort (Alawneh, et al., 2023).

Although *TWIST1* is highly expressed in cranial mesenchymal tissues, its transcriptional regulation may depend on inputs from more broadly expressed developmental factors. One such factor is *PITX2*, a paired-like homeodomain transcription factor essential for early craniofacial and dental morphogenesis. *PITX2* influences epithelial-mesenchymal signaling interactions, regulates downstream effectors of osteogenesis, and is required for neural crest cell survival and migration (Evans and Gage, 2005; Chawla, et al., 2016; Liu, et al., 2003; Li, et al., 2025). While classically viewed as an epithelial gene, *Pitx2* is also expressed in periocular and pharyngeal mesenchyme, where it participates in cranial base formation (Iwata, et al., 2012). In humans, *PITX2* mutations cause Axenfeld-Rieger syndrome, characterized by maxillary hypoplasia and dental anomalies, as well as prominent craniofacial features (Semina, et al., 1996).

Notably, *in silico* motif analysis identified rs2189000 within a highly conserved *PITX2* binding motif upstream of *TWIST1*. This raises the possibility that allelic variation at this site could disrupt transcriptional regulation of *TWIST1* by *PITX2*, providing a functional mechanism for the observed association with skeletal morphology. While *PITX2* and T*WIST1* have not been extensively studied in a common regulatory context, their spatial and temporal co-expression during craniofacial development suggests potential convergence in gene networks governing postnatal morphogenesis (Iwata, et al., 2012; Minoux, et al., 2017).

This study builds on our earlier genetic association work to interrogate the regulatory mechanisms underlying craniofacial growth variation. Based on this rationale, we hypothesize that rs2189000 is a functional noncoding variant that affects *TWIST1* expression by disrupting *PITX2*-mediated transcriptional activation, and *Twist1* disruption contributes to altered craniofacial phenotype during postnatal development, leading to malocclusion-related phenotypes. Additionally, by integrating deep sequencing of the *TWIST1* locus, functional characterization of enhancer activity, and phenotypic analysis of *Twist1* conditional knockout mice, we aim to delineate a developmental pathway linking common genetic variation to postnatal craniofacial dysmorphology.

## 2 Methods

### 2.1 Ethical approval and reporting guidelines

Human and animal research components of this study adhered to the STROBE and ARRIVE 2.0 checklists, respectively. All procedures were approved by the University of Iowa Institutional Review Board and IACUC.

### 2.2 Human study design

#### 2.2.1 Sample and phenotyping

A total of 277 untreated, non-syndromic Caucasian individuals (F = 195, M = 82; age 12–68, mean = 30) with malocclusion were included, as previously described (da Fontoura et al., 2015), and enriched by eight subjects (da Fontoura, et al., 2015). Skeletal classifications (Class I, II, III) were determined using established cephalometric criteria. Landmarking of lateral cephalometric images were performed using Dolphin Imaging® v11.5.04.35. Reliability were assessed via intraclass correlation coefficients (ICC >0.85) and magnification adjustments applied to analog films. Coordinates were exported for geometric morphometric (GM) analysis in MorphoJ (Klingenberg, 2011).

#### 2.2.2 Genotyping and sequencing

Genotyping of an additional 21 SNPs near the *TWIST1* locus was performed using KASPar competitive allele-specific PCR (KBioscience Ltd.) and Fluidigm 7900HT instrumentation. Sanger sequencing of an 84 kb region spanning *TWIST1* and *FERD3L* (7p21.1) was completed in 272 individuals using 31 overlapping primers designed with Primer 3.0, base-called with PHRED, assembled via PHRAP, scanned with POLYPHRED, and visualized in CONSED (Supplementary Figure S1). An additional 126 variants were tested for genotype-phenotype correlations.

#### 2.2.3 Genotype–phenotype correlation

Building on our prior work (da Fontoura, et al., 2015), we performed genotype–phenotype association analyses using the same pool of subjects and previously defined phenotypic traits derived from lateral cephalometric imaging. Additional SNPs identified by expanded sequencing were correlated with the craniofacial shape parameters from that original cohort. Among the 126 variants discovered, 54 common variants were selected based on allele frequency and genotyping completeness.

Multivariate linear regressions were conducted in Stata (StataCorp, College Station, TX), adjusting for age, sex, and image acquisition modality (analog film, digital radiograph, or CBCT). Craniofacial shape variables, defined through geometric morphometric analysis and principal component decomposition, were dependent variables for assessing genotype–phenotype associations. In addition, the type of skeletal class was evaluated as a categorical phenotype to assess the risk of developing skeletal malocclusion Class II or Class III compared to Class I.

### 2.3 Functional analyses

#### 2.3.1 Luciferase reporter assays

A 4.72 kb *TWIST1* enhancer fragment containing either wild-type or rs2189000 alleles were cloned upstream of a pTK-Luc vector (Promega). Five cell lines were used: HEPM (human mesenchymal), CHO, LS-8 (mouse oral epithelium), GMSM-K (human oral epithelium), and 293T. Transfections were carried out with Lipofectamine 2000 or PEI (Polysciences, cat# 23966-2). Luciferase activity was measured with the Dual-Luciferase® Reporter Assay Kit (Promega), normalized to β-galactosidase activity (Galacto-Light Plus, Tropix Inc.). Three to five independent replicates were performed for each condition, and independent, two-tailed t-tests were used to assess significance. Acknowledging the number of multiple comparisons, results were interpreted with caution, but no formal correction (e.g., Bonferroni) was applied due to hypothesis-driven design.

#### 2.3.2 Western blot

*TWIST1* protein detection was performed in HEPM cells using a mouse monoclonal *TWIST1* antibody (GXT, 1:1000) with GAPDH (Santa Cruz) as a loading control. Protein lysates (10 µg) were separated via SDS-PAGE, transferred to PVDF filters, and visualized with ECF substrate on a Typhoon 9410 imager (GE Healthcare).

#### 2.3.3 Chromatin immunoprecipitation (ChIP)

HEPM cells were cross-linked with 1% formaldehyde, lysed, and sonicated to produce 200–1000 bp DNA fragments. Immunoprecipitation was performed using anti-PITX2 (Capra Sciences, PA-1023) or IgG control. Enrichment of *TWIST1* enhancer fragments was assessed by PCR and quantified relative to input DNA. Three biological replicates were performed and reported as mean ± SEM.

### 2.4 Mouse model and experimental design

All animal protocols adhered to the University of Iowa Institutional Animal Care and Use Committee (IACUC) guidelines. *Twist1* conditional knockout mice were generated via a mesoderm-specific Cre-loxP strategy as described by (Chen et al., 2007). Mice were maintained on a mixed C57BL/6 background (Chen et al., 2007).

Experimental animals were euthanized at postnatal day 14 (P14) and day 21 (P21) via CO_2_ asphyxiation. Whole heads were fixed in 70% ethanol. Genotyping was performed on tail biopsy DNA using PCR amplification with validated primers.

Group sizes included - p21: WT (n = 7), *Twist1* ^*flox/+/Mesp1-Cre*^ (n = 6), *Twist1* ^*flox/flox/Mesp1-Cre*^ (n = 5) and p14: WT (n = 3), *Twist1 Twist1* ^*flox/+/Mesp1-Cre*^ (n = 3), *Twist1* ^*flox/flox/Mesp1-Cre*^ (n = 3).

#### 2.4.1 MicroCT imaging and preprocessing

Skull Heads (27) previously fixed in 70% ethanol were stored in saline water overnight. Group sizes included - p21: WT (n = 7), *Twist1* ^*flox/+/Mesp1-Cre*^ (n = 6), *Twist1* ^*flox/flox/Mesp1-Cre*^ (n = 5) and p14: WT (n = 3), *Twist1 Twist1* ^*flox/+/Mesp1-Cre*^ (n = 3), *Twist1* ^*flox/flox/Mesp1-Cre*^ (n = 3).

Heads were imaged using a SkyScan 1272 scanner (Bruker, Kontich, Belgium) with the following settings: 70 kVp, 142 μA, 0.5 mm Al filter, 18 μm voxel resolution, 0.3° rotation steps, and 3-frame averaging.

Image reconstruction was conducted using NRecon v1.7.1.0, followed by reorientation and beam hardening correction via DataViewer v1.5.2. DICOM conversion was completed with DicomCT v2.1. 3D renderings were visualized using CTVox v3.3.0.

#### 2.4.2 Landmark digitization and morphometric design

**FIGURE 1:**
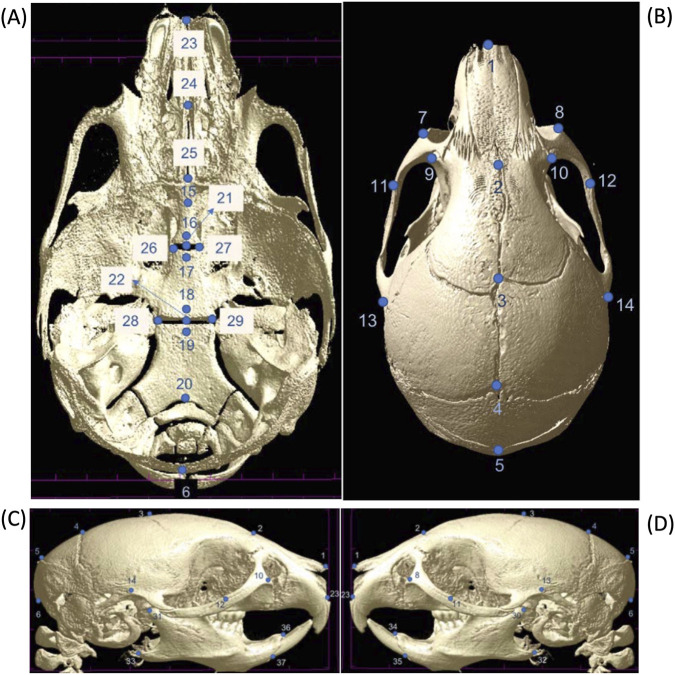
Three-dimensional cranial landmark configuration for morphometric analysis. **(A)** Ventral (cranial base) view of a representative mouse skull showing midline and bilateral landmarks along the premaxilla, palatine, sphenoid, and occipital regions. **(B)** Dorsal (calvarial) view showing landmarks distributed along the nasal, frontal, parietal, and interparietal bones and bilaterally at the zygomatic arches. **(C)** Lateral views of the right side of the skull depicting landmark placement across the snout, maxilla, mandible, orbit, and cranial vault. **(D)** Lateral views of the left side of the skull depicting landmark placement across the snout, maxilla, mandible, orbit, and cranial vault. Landmarks (n = 37) were recorded bilaterally and averaged across sides to account for natural symmetry, unless asymmetry was specifically analyzed. This configuration was used to calculate Euclidean distances and conduct geometric morphometric shape analysis.

Following reconstruction, 37 anatomically defined three-dimensional landmarks were manually digitized in 3D Slicer v4.11.0, guided by a validated murine craniofacial landmark protocol. Landmarks captured shape information across the cranial vault, cranial base, facial skeleton, and mandible. Bilateral landmarks were recorded on both sides, and symmetry was addressed by averaging where appropriate (Figures 1A–D; Supplementary Table S1).

Raw landmark coordinates of the right and left symmetric were submitted to a generalized Procrustes analysis (GPA). The Procrustes residuals were used in a principal component analysis (PCA) to capture the main components of symmetric variation (Klingenberg, 2011). Craniofacial deformation patterns were visualized using wireframes. Centroid size was calculated and used as a proxy for overall skull size. Group comparisons were interpreted descriptively, given the limited sample sizes and the multivariate nature of shape data.

#### 2.4.3 Linear distance analysis

A separate linear analysis was conducted to quantify discrete craniofacial dimensions based on the same landmark dataset. Anatomical distances were computed between predefined landmark pairs to assess features such as mandibular body length, ramus height, midfacial width, palatal length, and cranial base angle. For structures represented bilaterally, measurements were taken on both the left and right sides and averaged per specimen to account for asymmetry. All distances were analyzed using independent, two-tailed t-tests. A complete list of distances and their corresponding landmark definitions are presented in Supplementary Table S2.

## 3 Results

### 3.1 *TWIST1* genotype–phenotype correlations in malocclusion patients

**FIGURE 2:**
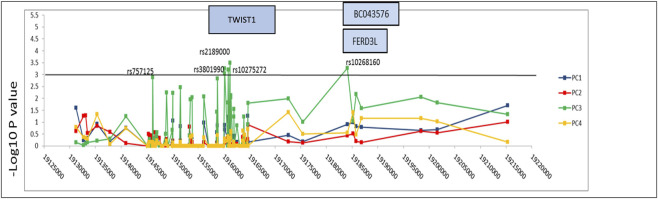
Genotype–phenotype associations across the *TWIST1* locus in individuals with malocclusion. Manhattan plot showing–log_10_(p) values for the association between 54 common SNPs spanning an 84 kb interval surrounding *TWIST1* and craniofacial shape variation, based on principal component (PC) scores derived from 3D landmark data. Results are shown for PC analysis, with PC3 (green line) displaying the strongest association signals. The peak signal corresponds to rs2189000 (P = 0.0003), located approximately 4.2 kb upstream of the *TWIST1* transcription start site, within a predicted enhancer region. Several neighboring SNPs in high linkage disequilibrium (D' = 1) with rs2189000 also reach significance (P < 0.001), forming a cluster of association centered at the *TWIST1* locus. The horizontal black line denotes a suggestive significance threshold at–log_10_(p) = 3.0. Nearby genes (*BC043576*, *FERD3L*) are shown, though association signals are specific to the regulatory interval upstream of *TWIST1*. PC3 shape variation, which reflects differences in mandibular ramus height and anterior cranial base inclination, is visualized in Supplementary Figure S2.

Genotype–phenotype correlation analysis across 277 individuals was focused on the 54 common variants identified within the 84 Kb interval spanning the *TWIST1* and *FERD3L* loci (chr7p21.1:19131207–19215314 bp). Multivariate linear regression analyses of PCs 1–4 derived from craniofacial cephalometric data confirmed that PC3 remained the most significantly associated phenotype, with the strongest signal observed for the noncoding SNP rs2189000 (P = 0.0003; Table 1; Figure 2). The effect size associated with rs2189000 was modest and showed substantial overlap with our prior candidate gene analysis, indicating consistency rather than amplification of the previously reported association (da Fontoura, et al., 2015). As previously described, PC3 captured shape variance related to anterior cranial base steepness and mandibular morphology (Supplementary Figure S2).

**TABLE 1:** Summary of top SNPs significantly associated with PC3 craniofacial shape variation. PC3 shape variation, which captures differences in mandibular ramus height and anterior cranial base inclination (see Supplementary Figure S2), showed significant associations (P < 0.001) with five common SNPs located upstream of *TWIST1*. The strongest association was observed for rs2189000 (P = 0.0003), located 3.9 kb upstream of the *TWIST1* transcription start site. All five SNPs are in strong linkage disequilibrium with rs2189000 (D' ≥ 0.95), and three lie within 3.6 kb of the gene. Minor allele frequencies (MAFs) are shown for both the 1000 Genomes reference population and the study cohort. RegulomeDB scores indicate potential regulatory function, with lower scores reflecting stronger evidence of transcription factor binding or regulatory activity.

Variant	Variant type/function	Location	MA = MAF in 1000 genomes	MA = MAF in study	Assoc. P Value	LD with rs2189000 (D'/R2)	Regulome DB score
rs3801991	Variant 2.8 Kb upstream of *TWIST1*	19160177	A = 16.5%	A = 12.4%	0.0009	1/0.95	5
rs3801990	Variant 2.8 Kb upstream of *TWIST1*	19160183	G = 16.5%	G = 12.6%	0.0005	1/0.95	5
rs10275272	Variant 3.6 Kb upstream of *TWIST1*	19160897	T = 28%	T = 14.4%	0.0006	1/1	4
rs2189000	Variant 3.9 Kb upstream of *TWIST1*	19161218	G = 27%	G = 16.6%	0.0003	1/1	5
rs10268160	Non-codding transcript variant	19184059	G = 32%	G = 20%	0.0005	1/0.814	4

Five associated SNPs demonstrated strong linkage disequilibrium (D’ = 1) with each other, suggesting they likely capture the same association signal (Table 1). Notably, annotations using Decipher and Copy Number Variant tracks (UCSC browser) indicated that individuals with structural variation in the genomic region around these SNPs present with craniofacial anomalies resembling SCS.

Additionally, logistic regression of categorical analysis of skeletal Class II and Class III malocclusion showed that the risk of Class III (vs. Class I) was significantly reduced in carriers of the minor allele of rs117390026 (OR = 0.17, P = 0.04). However, this signal did not surpass multiple-testing correction.

### 3.2 Functional validation: *PITX2* regulates *TWIST1* via rs2189000

**FIGURE 3:**
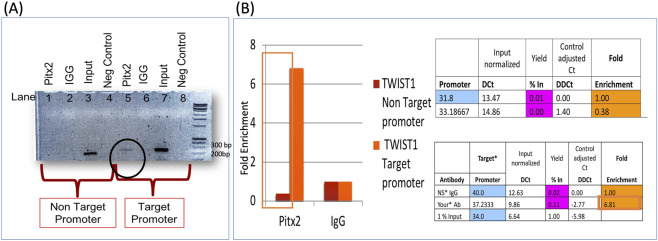
Functional validation of PITX2 regulation of TWIST1 through the rs2189000 enhancer. **(A)** Chromatin immunoprecipitation (ChIP) assay using PITX2 antibody in HEPM cells demonstrates selective enrichment of the *TWIST1* upstream region containing the rs2189000-A allele. PCR amplification of the pulled-down DNA shows a clear band in PITX2 antibody samples (lanes 5 and 6), but not in IgG controls (lanes 3 and 4), confirming *PITX2* binding at the enhancer locus. **(B)** Quantitative analysis of ChIP-qPCR enrichment in HEPM cells reveals ∼6.8-fold enrichment of the rs2189000-A region in PITX2 antibody samples compared to IgG control. Data show ΔCt values (blue), corresponding p-values (pink), and fold enrichment (orange). These results confirm that *PITX2* directly occupies the rs2189000-containing enhancer sequence in mesenchyme-derived cells.

The rs2189000 SNP lies ∼4.2 kb upstream of the *TWIST1* transcription start site, within a conserved predicted *PITX2* binding motif. Chromatin immunoprecipitation ChIP assays in HEPM cells showed ∼6-fold enrichment of *TWIST1* sequence containing the rs2189000-A allele over IgG, indicating *PITX2* occupancy at this enhancer site in mesenchyme-derived cells (Figures 3A,B). *PITX2* binding to the *TWIST1* enhancer was confirmed by qPCR amplification of the target promoter region (circled in Figure 3A), with quantitative enrichment shown in Figure 3B.

**FIGURE 4:**
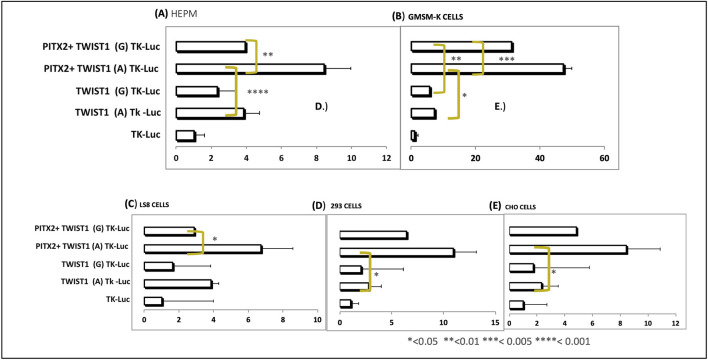
*PITX2* activates transcription via the rs2189000-A allele in luciferase reporter assays. **(A–E)** Luciferase reporter assays were conducted in five independent cell lines. **(A)** HEPM, **(B)** GMSM-K, **(C)** LS8, **(D)** 293, and **(E)** CHO cells, comparing the ancestral (A) and risk (G) alleles of rs2189000. In all cell types, *PITX2* significantly activated transcription through the rs2189000-A allele. In HEPM cells, a 4.62-fold increase in luciferase activity was observed when compared to the empty vector (P = 0.0007). Mutation of the site to the rs2189000-G allele abolished *PITX2*-mediated activation. Bars represent mean ± SEM. Statistical comparisons were performed between alleles; significance is indicated as *P < 0.05; **P < 0.01; ***P < 0.005; ****P < 0.001.

Luciferase reporter assays confirmed that *PITX2* activates transcription via the rs2189000-A (ancestral) allele. Transfection of Pitx2 in HEPM cells activated the A allele at 8.62-fold, compared to the G allele with Pitx2 and A allele without Pitx2 at 4-fold (P = 0.0007). Mutating the site to the rs2189000-G allele significantly reduced *PITX2*-mediated activation (Figures 4A–E). These findings were consistent across four additional cell lines.

### 3.3 Geometric morphometric analysis reveals genotype-specific shape differences in mice

**FIGURE 5:**
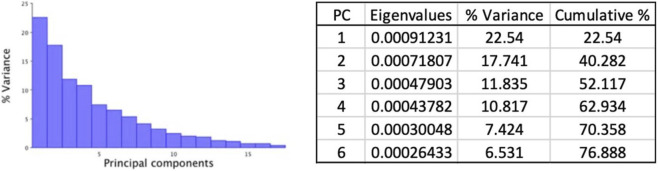
Screen plot illustrating the variance explained by each principal component (PC).

To investigate postnatal craniofacial shape variation in *Twist1* conditional knockout mice, we performed a three-dimensional GM analysis at postnatal day 21 (P21), using a landmark-based GM approach (Lieberman, et al., 2000). GM analysis of P21 mouse skulls identified six principal components explaining 76.8% of the total craniofacial shape variance: PC1 (22.5%), PC2 (17.7%), PC3 (11.8%), PC4 (10.8%), PC5 (7.4%), PC6 (6.5%) (Figure 5).

- PC1: captured doming of the cranial vault and shortening of the cranial base; *Twist1* ^*flox/+/Mesp1-Cre*^, *Twist1* ^*flox/flox/Mesp1-Cre*^ mice showed extreme positive PC1 values, indicating increased vault height and steep anterior cranial base flexion.
- PC2: separated groups based on mandibular ramus angle and gonial inclination. WT mice showed anterior mandibular translation, while mutants showed posterior retrusion and reduced body size.
- PC3: highlighted flexion and asymmetry in anterior–posterior cranial dimensions but did not strongly distinguish genotypes.

**FIGURE 6:**
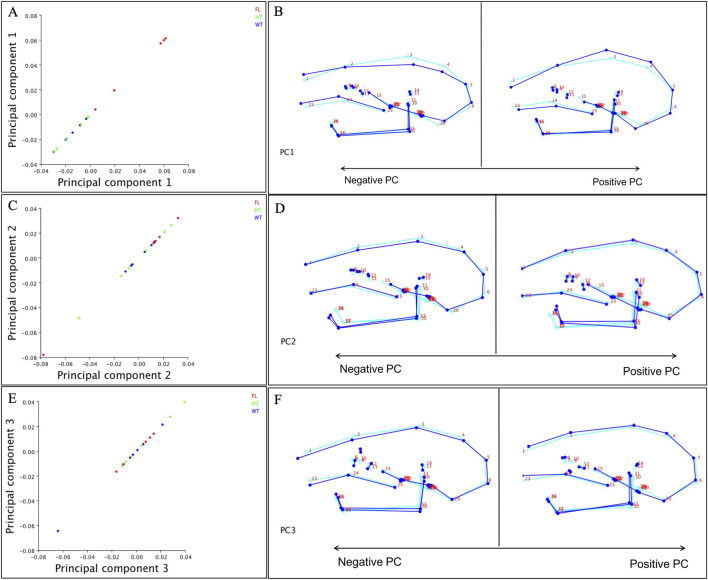
Principal component analysis (PCA) of craniofacial shape at P21 reveals genotype-specific trends. **(A,C, E)** Scatterplots of PC1, PC2, and PC3 scores for wild-type (WT, blue), heterozygous (green), and Twist1 floxed homozygous mutants (red). While genotypic overlap is present in PC space, the floxed group shows a trend toward the positive extremes on all three axes, indicating consistent craniofacial deviations. **(B,D,F)** Wireframe visualizations illustrate shape changes associated with the negative and positive extremes of PC1–PC3 axes. PC1: Floxed mice display pronounced cranial vault doming and shortening of the cranial base. PC2: Marked reduction in mandibular length, retrusion of the mandibular body, and an increased gonial angle are observed in the floxed group. PC3: Floxed animals show anterior–posterior compression of midfacial structures and subtle craniofacial asymmetries.

Overall, genotype-specific differences were observed at P21, suggesting that craniofacial dysmorphology progresses with postnatal growth. Shape differences between wild-type (WT) and homozygous mutants were more pronounced than between WT and heterozygous animals, with the heterozygous group often exhibiting intermediate morphology. Wireframe visualizations illustrate shape changes at the negative and positive extremes of the PC1–PC3 axes (Figure 6).

PC1 primarily captured variation in cranial vault curvature, anterior cranial base length, and palatal inclination. Homozygous mutants showed positive PC1 scores, reflecting an exaggerated doming of the skull, reduced cranial base and palatal length, and downward rotation of the palate. In contrast, WT animals exhibited negative PC1 values, associated with flatter cranial vaults and elongated anterior-posterior dimensions.

PC2 described changes in mandibular morphology, particularly affecting the ramus height, gonial angle, and mandibular body projection. WT specimens displayed anterior and inferior mandibular translation, while homozygous mutants showed posterior displacement and a shortening of the mandibular body, indicative of altered mandibular growth trajectory.

Together, these results demonstrate that *Twist1* deletion in cranial mesoderm leads to progressive and region-specific alterations in craniofacial shape, affecting the cranial base, cranial vault, palate, and mandible during postnatal growth. These differences increased from P14 to P21, indicating progressive divergence with postnatal growth. Although some genotype overlap occurred in PC scatterplots, the shape extremes visualized by wireframes suggested biologically relevant and localized shifts, particularly in vault curvature, palatal rotation, and mandibular geometry.

### 3.4 Linear craniometrics confirm altered growth in mutant mice

Linear craniofacial dimensions were assessed across the full cohort of mice using Euclidean distances between predefined 3D landmarks captured from microCT reconstructions. As outlined in Supplementary Table S1, these landmarks allowed for precise quantification of anterior–posterior (A–P), vertical, and transverse skeletal dimensions across genotypic groups. A complete list of distances and their corresponding landmark definitions is presented in Supplementary Table S2.

#### 3.4.1 Anterior–posterior measurements

Overall skull length, defined by the distance between landmarks 1 and 6, was significantly reduced in both *Twist1* ^*flox/flox/Mesp1-cre*^ and *Twist1* ^flox/+/Mesp1−cre^ mice compared to WT controls at P14 and P21. The reduction in skull length was primarily attributed to shortening of the anterior cranial base, specifically in the pre-sphenoid and basi-sphenoid regions. In contrast, the basioccipital bone length (landmarks 19–20) did not change significantly at P21. However, at P14, all three cranial base segments exhibited significant shortening.

The most pronounced A–P difference was observed in the maxillary region at P21, between landmarks 24 and 25 (premaxilla to caudal palatine bone), with a highly significant p-value of 0.00005. While similar differences were noted at P14, statistical significance was only observed between *Twist1* ^flox/flox/Mesp1−cre^ and WT (p = 0.0359). Additional reductions in premaxilla length (23–24) and total maxilla length (23–25) were significant across all genotypic comparisons.

Reductions in the cranial vault and facial dimensions were also evident at P21. These included landmark intervals from nasion to bregma (2–3), nasion to ophistion (2–6), nasale to caudal palatine (1–25), and junctions at the interparietal and occipital bones (4–5 to 5–6). At P14, significant differences were also noted in dimensions spanning from nasale to nasion (1–2), nasion to bregma (2–3), nasion to ophistion (2–6), and nasale to ophistion (1–6).

The entire cranial base length (15–20) and its subcomponents—including pre-sphenoid (15–16) and basi-sphenoid (17–18) regions—were consistently reduced in mutants at both developmental stages. In contrast, no significant difference was found in the basioccipital segment (19–20), particularly at P21.

#### 3.4.2 Vertical measurements

Vertical measurements were taken using mid-sagittal craniofacial landmarks. At P21, *Twist1* ^flox/flox/Mesp1−cre^ mice exhibited significant reductions in cranial vault height, particularly in the region between bregma and the intersphenoid synchondrosis (3–21). Additionally, facial height (2–24) and posterior cranial height (5–20) were significantly decreased at both P14 and P21.

#### 3.4.3 Mandibular measurements

Four landmarks were placed on each side of the mandible to evaluate mandibular growth: at the condyle, mandibular angle, and upper and lower incisor roots. Significant changes were detected in both the A–P and transverse dimensions. The most substantial A–P reductions at P21 were observed in mandibular body length, notably between landmarks 32–35 (left side) and 33–37 (right side). Additional differences were seen in the upper mandibular body between landmarks 30–34 and 31–36.

Vertical mandibular measurements—spanning from condyle to angle (30–32 and 31–33)—did not differ significantly between genotypes. However, transverse measurements demonstrated notable narrowing in the *Twist1flox/flox/Mesp1-cre* group at P21. Inter-condylar width (30–31) and inter-angular width (32–33) were significantly reduced compared to WT and heterozygous littermates. Interestingly, no significant transverse mandibular differences were observed at P14, suggesting a postnatal onset of this phenotype.

In summary, Twist1-deficient mice displayed significant and progressive reductions in craniofacial dimensions across all three spatial planes—A–P, vertical, and transverse—by P21. These alterations mirror findings from geometric morphometric analyses and highlight the critical role of Twist1 in regulating coordinated postnatal craniofacial growth.

### 3.5 Premature synchondrosis closure correlates with phenotypic severity

**FIGURE 7:**
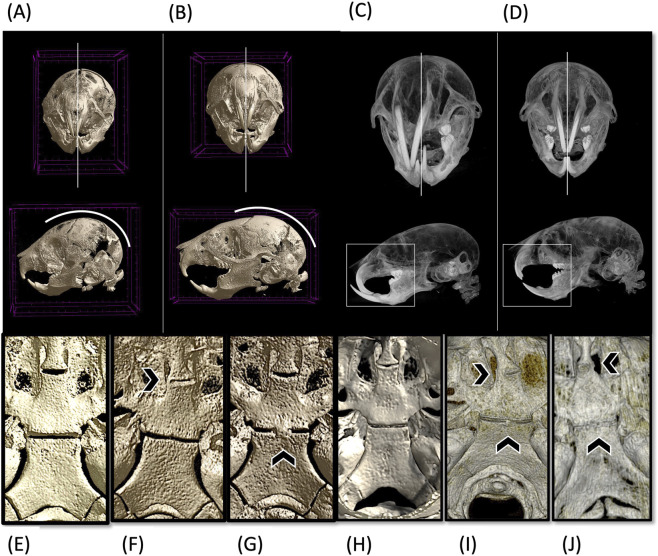
Craniofacial malformations and premature synchondrosis ossification in *Twist1* mutant mice. **(A,C)** μCT reconstructions of representative mutant (*Twist1* ^flox/flox/Mesp1Cre^) mice at postnatal day **(A)** 14 and **(C)** P21, showing increased cranial vault doming, shortened snouts, and facial asymmetry. By P21, several mutants exhibit severe anterior-posterior shortening and prominent malocclusion (“elephant tusk” phenotype). **(B,D)** Control wild-type (WT) specimens at P14 **(B)** and P21 **(D)** display a standard craniofacial phenotype, with patent sutures and symmetrical facial structures. **(E–G)** μCT reconstructions of the cranial base at P14 and **(H–J)** μCT reconstructions of the cranial base at P21. **(E)** Control showing patent intersphenoid (ISS) and spheno-occipital synchondroses (SOS). **(F,G)** Mutant specimens demonstrate early ossification of synchondroses, with bony bridging and partial fusion evident at both ISS and SOS, respectively. **(H)** Control with persistent synchondrosis patency. **(I,J)** Mutant specimens showing advanced ossification with mineralized callus formation at the ISS and complete fusion of the SOS, contributing to altered skull base growth. Scale bars: 1 mm.

The craniofacial phenotype of *Twist1* ^flox/flox/Mesp1−cre^ and Twist1^flox/+/Mesp1−cre^ mice is observed in association with the patency status of cranial base synchondroses. To evaluate postnatal craniofacial morphology, P14 and P21 mouse heads were analyzed via high-resolution microCT. This analysis revealed a previously uncharacterized craniofacial phenotype between *Twist1*-deficient and WT mice (Figures 7A–J).

Notably, premature fusion of the calvarial sutures, including the coronal suture, was observed as early as postnatal week one (data not shown). By P14, *Twist1* ^flox/flox/Mesp-cre^ and *Twist1* ^flox/+/Mesp1−cre^ mice exhibited snout deviation and mild facial asymmetry (Figure 7A). These features became more pronounced by P21 (Figure 7C), with greater snout deviation, increased asymmetry, and overt malocclusion. In cases where facial asymmetry was not evident, mutant mice instead showed marked snout shortening and enhanced cranial vault doming.

Interestingly, microCT imaging revealed the presence of small bony outgrowths bridging the intersphenoid synchondrosis (ISS) rostro-caudally as early as P14, with similar structures occasionally observed at the spheno-occipital synchondrosis (SOS) (Figures 7F,G). By P21, these bridges had developed into mineralized calluses that partially or completely obliterated the ISS in several specimens. In many cases, the left and right halves of a given synchondrosis exhibited asymmetric mineralization patterns, suggesting lateralized differences in fusion timing and ossification dynamics (Figures 7I,J).

The SOS, in particular, displayed a distinctive mineralization trajectory, with bony extensions emanating from the central basisphenoid region toward the basioccipital bones. These abnormal ossification patterns were consistently observed at both P14 and P21, reinforcing the notion that Twist1 deficiency disrupts synchondrosis regulation and contributes to the observed craniofacial dysmorphology.

## 4 Discussion

Craniofacial morphology results from the integrated actions of developmental patterning, transcriptional regulation, and tissue growth across embryonic and postnatal stages (Tseng and Crump, 2023). *TWIST1*, a basic helix–loop–helix transcription factor, plays an essential role in mesenchymal lineage specification and the maintenance of cranial suture patency during skull development (Bourgeois, et al., 1998; Johnson, et al., 1998; Chen and Behringer, 1995). While loss-of-function mutations in *TWIST1* are known to cause SCS, a craniosynostosis disorder with cranial abnormalities (Wilkie, et al., 1995; Alawneh, et al., 2023), the mechanisms by which common noncoding variants at this locus contribute to craniofacial variation in non-syndromic populations remain largely unexplored.

This study builds upon our prior human genetic analysis, which first identified the common SNP rs2189000 located upstream of *TWIST1* as significantly associated with a principal component of craniofacial shape variation, capturing mandibular ramus height, mandibular body length, and anterior cranial base inclination in our subjects. These traits are central to sagittal jaw discrepancies and are commonly implicated in malocclusion (Sanggarnjanavanich, et al., 2014; Gong, et al., 2016). Notably, the shape axis defined by PC3 parallels several cephalometric features characteristic of SCS, including mandibular deficiency and cranial base steepness, suggesting that common noncoding variants may phenocopy aspects of syndromic phenotypes in a dosage-sensitive manner.

In our extended cohort of 277 non-syndromic individuals with malocclusion, we identified rs2189000 as the top-associated SNP within the *TWIST1* interval (da Fontoura, et al., 2015). This common variant demonstrated a strong association with craniofacial shape variation, particularly affecting mandibular ramus height, mandibular body length, and anterior cranial base inclination. Sequencing of the full *TWIST1* interval did not identify any coding mutations, reinforcing the hypothesis that the phenotypic effects of rs2189000 arise from regulatory rather than structural gene changes. In addition to rs2189000, four other SNPs in strong linkage disequilibrium (D' = 1) also showed significant associations with PC3 (P < 0.001). Importantly, non-coding mutations were detected in *TWIST1* across the sequenced cohort. Annotation of the non-coding variants (SNV) of *TWIST1* was reported in patients with SCS, further supporting a regulatory role in craniofacial development and emphasizing the importance of screening the 5′ UTR in clinically diagnosed patients (Zhou, et al., 2018).

To explore the mechanism by which rs2189000 might alter *TWIST1* expression, we performed *in silico* transcription factor binding analysis. We identified that rs2189000 disrupts a highly conserved consensus DNA binding motif for *PITX2*, a paired-like homeodomain transcription factor essential for craniofacial morphogenesis, asymmetry, and odontogenesis (Liu et al., 2003; Evans and Gage, 2005; Cao(Liu, et al., 2003; Evans and Gage, 2005; Cao, et al., 2013). Luciferase assays in five independent cell lines—including human embryonic palatal mesenchymal (HEPM) cells, demonstrated that *PITX2* robustly activates transcription through the ancestral allele, but not the risk (G) allele of the enhancer. Chromatin immunoprecipitation was performed in HEPM cells homozygous for the rs2189000-A allele to assess *PITX2* occupancy at the *TWIST1* upstream enhancer and confirmed that *PITX2* binds the rs2189000-containing enhancer sequence in HEPM cells, with approximately six-fold enrichment over IgG controls, directly validating the enhancer’s putative function *in vitro*. Although we did not perform allele-specific ChIP, the loss of *PITX2*-mediated enhancer activation was functionally validated through luciferase assays using the risk (G) allele.

While our *in vitro* assays support *PITX2* as a transcriptional activator of *TWIST1* through the rs2189000 enhancer, we acknowledge the well-established spatial separation between *PITX2* and *TWIST1* expression domains, specifically, *PITX2* being enriched in craniofacial epithelia and *TWIST1* in cranial mesenchyme. This has raised valid concerns about the physiological relevance of a direct regulatory interaction *in vivo*. However, transcriptomic and gene expression atlas data suggest that *Pitx2* is transiently expressed in cranial neural crest and mesoderm-derived mesenchyme during early craniofacial development, particularly during palate and jaw formation, during stages when mesenchymal–epithelial crosstalk is prominent (Chawla, et al., 2016; Minoux, et al., 2017; Franco and Aranega, 2018; Evans and Gage, 2005). We interpret this not as evidence of a stable *PITX2*–*TWIST1* axis, but as supporting a temporally restricted regulatory event that could plausibly influence enhancer activation during critical windows of craniofacial development. Thus, the *PITX2* experiments presented here serve as a functional test of the rs2189000 variant’s putative regulatory potential *in vitro*. Future studies will be needed to identify whether *PITX2* or other transcription factors engage this enhancer *in vivo* within the mesenchymal lineage.

To evaluate the physiological relevance of *TWIST1* dysregulation, we examined the craniofacial phenotypic variations of a *Mesp1* ^*Cre*^ conditional knockout model targeting cranial mesoderm. While the mandibular skeleton is derived primarily from cranial neural crest cells, the cranial base originates from cranial mesoderm and plays a critical role in establishing mandibular position through its influence on occlusal plane angulation, condylar orientation, and vertical facial proportions. Prior studies confirm that Mesp1^Cre^ efficiently targets early cranial mesoderm without affecting epithelial or neural crest-derived domains (Bildsoe, et al., 2013; Chen, et al., 2007). Our decision to employ *Mesp1* ^*Cre*^ mediated deletion of *Twist1* was therefore based on the hypothesis that early perturbations in cranial base development could drive secondary alterations in mandibular shape and spatial orientation. This rationale is supported by our human genotype–phenotype analyses, in which the rs2189000 risk allele was associated with reduced ramus height, increased mandibular body length, and a steep anterior cranial base, features consistent with cranial base–driven Class III skeletal relationships. Although Wnt1-Cre–mediated deletion of Twist1 has been shown to cause profound disruptions in mandibular development, the *Mesp1* ^*Cre*^ model enabled assessment of more subtle, postnatal morphologic changes spanning both cranial base and mandibular structures. This approach thus captures a distinct axis of craniofacial variation that is directly relevant to the pathogenesis of malocclusion.

Postnatal morphometric analyses of high-resolution microCT scans of mice heads at postnatal days 14 and 21 revealed significant genotype-dependent craniofacial differences. PCA of three-dimensional landmark data demonstrated separation from wild-type mice along PCs 1 and 2, reflecting differences in cranial base angle, cranial vault doming, and mandibular projection. Wireframe comparisons further highlighted biologically meaningful variation in these axes. While scatterplots showed some group overlap, wireframes revealed consistent shape trends distinguishing genotypes. Notably, homozygous mutants exhibited a domed cranial vault, shortened cranial base, and posteriorly rotated mandible. Specifically, wild-type mice exhibited flatter cranial vaults, longer cranial bases, and more prominent mandibular extension, whereas homozygous mutants presented with domed calvaria, shortened cranial bases, and posteriorly rotated mandibles. These differences are visualized in wireframe overlays that contrast the extremes of PC1 between genotypes, despite partial overlap in scatterplots. Notably, the craniofacial variations observed in mutant mice closely resemble those observed in the human cohort, underscoring the conserved craniofacial phenotypic effects of *TWIST1* dysregulation across species.

Linear measurements confirmed these findings, demonstrating statistically significant reductions in skull length, cranial base length, palatal dimensions, and mandibular body length in mutant mice compared to controls. These phenotypes closely mirror cephalometric patterns documented in SCS patients, who present with shortened ramus height, steep mandibular planes, and anterior cranial base defects (Lonsdale, et al., 2019; Evans and Christiansen, 1976).

A particularly novel finding was the premature ossification of cranial base synchondroses in mutant mice. In wild-type animals, the intersphenoid (ISS) and spheno-occipital (SOS) synchondroses remain patent into adolescence, serving as critical growth centers for anterior cranial base elongation (Vora, et al., 2015; Hallett, et al., 2025; Funato, 2020; Wei, et al., 2017). However, *Twist1*-deficient mice exhibited partial or complete fusion of these synchondroses by P21, as revealed by microCT and Alizarin Red staining. These fusions were accompanied by cranial vault doming, snout asymmetry, and malocclusion, including the distinctive “elephant tusk” appearance previously described in *Twist1* deletion models (Bourgeois, et al., 1998). Although incisor overgrowth is a common confounder in rodent malocclusion models, we attribute these skeletal deformities to underlying asymmetries and premature fusion events that disrupt postnatal growth trajectories.

These findings highlight the capacity of common regulatory variants to contribute to complex traits and bridge the gap between syndromic and non-syndromic presentations. While rs2189000 does not cause disease, it contributes to a spectrum of craniofacial variation that overlaps with *TWIST1* loss-of-function syndromes. This concept of “intermediate phenotypes” is particularly informative for understanding gene dosage effects in craniofacial development (Sharma, et al., 2013). Moreover, our results reinforce the importance of considering noncoding variation in genetic studies of malocclusion and jaw development. Despite most clinical genetics focusing on coding mutations, regulatory elements may underlie unexplained cases of craniofacial asymmetry or growth anomalies.

Our study also illustrates the value of integrating genomic, functional, and phenotypic datasets across species. While the *PITX2–TWIST1* regulatory axis was validated *in vitro*, future studies should test *in vivo* enhancer occupancy using ChIP-seq or CRISPRi strategies in embryonic mesenchymal populations. Additionally, given that the *TWIST1* locus is enriched for regulatory elements based on FANTOM5 and ENCODE annotations, further chromatin conformation and enhancer–promoter interaction mapping will be necessary to define the whole regulatory architecture underlying craniofacial growth.

This study has limitations. While the knock-in model did not contain an rs2189000 directly, our integrated data, combining genotype–phenotype associations, allele-specific luciferase assays, and conditional *Twist1* deletion, support the functional relevance of this site. Future studies employing precise genome editing to model this variant *in vivo* will be necessary to definitively test its phenotypic consequences.

Furthermore, the mouse sample size, especially in homozygous mutants, restricts statistical power. While appropriate statistical tests were applied, the qualitative nature of morphometric findings and the variability across individuals underscore the need for larger cohorts. Additionally, the *PITX2–TWIST1* regulatory axis was validated *in vitro*; future studies should test *in vivo* enhancer occupancy using ChIP-seq or CRISPRi strategies in embryonic mesenchymal populations. Given that the *TWIST1* locus is enriched for regulatory elements based on FANTOM5 and ENCODE annotations, further chromatin conformation and enhancer–promoter interaction mapping will be necessary to define the whole regulatory architecture underlying craniofacial growth.

In conclusion, we provide evidence that a common noncoding variant upstream of *TWIST1* alters enhancer activity *in vitro* experiments, suggesting a possible regulatory mechanism contributing to variation in jaw relations and cranial base morphology These results extend the functional impact of *TWIST1* beyond syndromic contexts and demonstrate that noncoding regulatory variation can influence craniofacial structure in non-syndromic individuals. This work offers a framework for future studies aiming to unravel the complex genetic underpinnings of malocclusion and craniofacial diversity.

## Data Availability

The raw data supporting the conclusions of this article will be made available by the authors, without undue reservation.
