# Supplementary material for: Exploring the role of TWIST1 in malocclusion and craniofacial morphology
Source: Front Physiol. 2026 Feb 17;17:1749243. doi: 10.3389/fphys.2026.1749243 (PMC12952720; doi:10.3389/fphys.2026.1749243)

Supplementary Material

**Supplementary Table 1. Description of the 37 landmarks digitized on mice head micro CT images.**

| Landmark Number | Landmark |
| --- | --- |
| 1 | Nasale |
| 2 | Nasion |
| 3 | Bregma |
| 4 | Intersection of parietal bones with anterior aspect of interparietal bone at midline |
| 5 | Intersection of interparietal bone with occiptal bone at midline |
| 6 | Ophistion |
| 7 | Anterior notch of zigimatic-left |
| 8 | Anterior notch of zigimatic-right |
| 9 | Intersection of frontal process of maxilla with frontal and lacrimal bones, left side |
| 10 | Intersection of frontal process of maxilla with frontal and lacrimal bones, right side |
| 11 | Intersection of zygomatic process of maxilla with zygoma (jugal), superior surface, left side |
| 12 | Intersection of zygomatic process of maxilla with zygoma (jugal), superior surface, right side |
| 13 | Zygomatic root connecting scamosal body-Left |
| 14 | Zygomatic root connecting scamosal body-right |
| 15 | Pre-sphenoid bone at sagital plane |
| 16 | Anterior ISS at mid sagital plane |
| 17 | Posterior ISS at mid sagital plane |
| 18 | Anterior SOS at mid sagital plane |
| 19 | Posterior SOS at mid sagital plane |
| 20 | Caudal point of basi-occiptal bone |
| 21 | ISS at mid sagital plane |
| 22 | SOS at mid sagital plane |
| 23 | Anterior most point of upper alveolus of upper incisor |
| 24 | Intersection maxilla and pre-maxilla |
| 25 | Caudal point of palatine bone |
| 26 | Left margin of ISS horizontal view |
| 27 | Right margin of ISS horizontal view |
| 28 | Left margin of SOS horizontal view |
| 29 | Right margin of SOS horizontal view |
| 30 | Posterior most point on condile-Left side |
| 31 | Posterior most point on condile- Right side |
| 32 | Tip of mandilular angle- Left side |
| 33 | Tip of mandilular angle- Right side |
| 34 | Superior rim of the bone-tooth junction of the lower incisor- Left side |
| 35 | Inferior rim of the bone-tooth junction of the lower incisor- Left side |
| 36 | Superior rim of the bone-tooth junction of the lower incisor- Right side |
| 37 | Inferior rim of the bone-tooth junction of the lower incisor- Right side |

**Supplementary Table 2. List of linear measurements used in this study.**

| Type | Landmarks | Description |
| --- | --- | --- |
| Anterior-posterior | 1-2 | Length of nasal bone |
| Anterior-posterior | 2-3 | Length of frontal bone |
| Anterior-posterior | 3-4 | Length of parietal bone |
| Anterior-posterior | 4-5 | Length of interparietal bone |
| Anterior-posterior | 5-6 | Length of occipital bone |
| Anterior-posterior | 1-6 | Skull length |
| Anterior-posterior | 2-6 | Cranial length |
| Anterior-posterior | 1-25 | Facial length |
| Anterior-posterior | 2-15 | Anterior cranial base length |
| Anterior-posterior | 15-16 | Length of pre-sphenoid bone |
| Anterior-posterior | 15-20 | Posterior cranial base length |
| Anterior-posterior | 16-17 | Length of ISS |
| Anterior-posterior | 17-18 | Length of basi-sphenoid bone |
| Anterior-posterior | 18-19 | Length of SOS |
| Anterior-posterior | 19-20 | Length of basi-occipital bone |
| Anterior-posterior | 23-24 | Length of pre-maxilla |
| Anterior-posterior | 23-25 | Palatal length |
| Anterior-posterior | 24-25 | Posterior palate length |
| Anterior-posterior | 30-34 | Upper mandibular length, left side |
| Anterior-posterior | 31-36 | Upper mandibular length, right side |
| Anterior-posterior | 32-35 | Lower mandibular length, left side |
| Anterior-posterior | 33-37 | Lower mandibular length, right side |
| Transversal | 7-8 | Anterior zygomatic width -facial width |
| Transversal | 9-10 | Inter-orbital width |
| Transversal | 11-12 | Inter-zygomatic arches width |
| Transversal | 13-14 | Zygomatic width - skull width |
| Transversal | 26-27 | Width of ISS |
| Transversal | 28-29 | Width of SOS |
| Transversal | 30-31 | Inter-condile width |
| Transversal | 32-33 | Mandibular width |
| Vertical | 2-24 | Facial height |
| Vertical | 3-21 | Anterior-cranial height |
| Vertical | 4-22 | Mid-cranial height- cranial height |
| Vertical | 5-20 | Posterior-cranial height |
| Vertical | 30-32 | Mandibular height, left side |
| Vertical | 31-33 | Mandibular height, right side |

**Supplementary Table 3. Linear Craniometric Measurements in P14 and P21 Mice Across Genotypes**

This table summarizes the Euclidean distances between anatomically defined cranial and mandibular landmarks in wild-type, heterozygous *Twist1^f^*^lox/+Mesp1Cre^, and homozygous mutant *Twist1*^flox/floxMesp1Cre^ mice at postnatal day 14 (P14) and day 21 (P21). Measurements include anterior–posterior (A–P) skull length, cranial base length, cranial vault height, palatal width, and mandibular dimensions. Statistically significant differences (p < 0.05) are indicated for each intergroup comparison, highlighting genotype-dependent changes in craniofacial growth. Bolded values indicate the most pronounced differences, particularly in anterior cranial base and mandibular body length at P21.

| Type | Landmarks | Description | P14 Twist1 Flox/Flox vs.  WT | P14 Twist1 Flox/+  vs.  WT | P14 Twist1 Flox/Flox  vs.  Twist1 Flox/+ | P21 Twist1 Flox/Flox vs.  WT | P21 Twist1 Flox/+  vs.  WT | P21 Twist1 Flox/Flox  vs.  Twist1 Flox/+ |
| --- | --- | --- | --- | --- | --- | --- | --- | --- |
| Anterior-posterior | 1-2 | Length of nasal bone | 0.311 | 0.339 | 0.397 | 0.09043 | 0.28785 | 0.24860 |
| Anterior-posterior | 2-3 | Length of frontal bone | 0.035 | 0.203 | 0.126 | 0.00042 | 0.45958 | 0.00849 |
| Anterior-posterior | 3-4 | Length of parietal bone | 0.387 | 0.302 | 0.244 | 0.24182 | 0.45763 | 0.38564 |
| Anterior-posterior | 4-5 | Length of interparietal bone | 0.393 | 0.004 | 0.034 | 0.48761 | 0.40985 | 0.38961 |
| Anterior-posterior | 5-6 | Length of occipital bone | 0.092 | 0.314 | 0.005 | 0.04984 | 0.16752 | 0.12522 |
| Anterior-posterior | 1-6 | Skull length | 0.015 | 0.120 | 0.006 | 0.00042 | 0.24731 | 0.00536 |
| Anterior-posterior | 2-6 | Cranial length | 0.003 | 0.101 | 0.004 | 0.00050 | 0.45409 | 0.00192 |
| Anterior-posterior | 1-25 | Facial length | 0.031 | 0.099 | 0.052 | 0.00246 | 0.35935 | 0.01226 |
| Anterior-posterior | 2-15 | Anterior cranial base length | 0.031 | 0.062 | 0.079 | 0.16032 | 0.25338 | 0.25797 |
| Anterior-posterior | 15-16 | Length of pre-sphenoid bone | 0.020 | 0.056 | 0.022 | 0.00050 | 0.36210 | 0.00284 |
| Anterior-posterior | 15-20 | Posterior cranial base length | 0.332 | 0.457 | 0.464 | 0.00134 | 0.48734 | 0.00527 |
| Anterior-posterior | 16-17 | Length of ISS | 0.010 | 0.209 | 0.004 | 0.37835 | 0.27316 | 0.40575 |
| Anterior-posterior | 17-18 | Length of basi-sphenoid bone | 0.320 | 0.013 | 0.008 | 0.00167 | 0.24384 | 0.00898 |
| Anterior-posterior | 18-19 | Length of SOS | 0.011 | 0.248 | 0.018 | 0.20809 | 0.08399 | 0.04232 |
| Anterior-posterior | 19-20 | Length of basi-occipital bone | 0.010 | 0.071 | 0.009 | 0.26386 | 0.31163 | 0.37402 |
| Anterior-posterior | 23-24 | Length of pre-maxilla | 0.031 | 0.484 | 0.109 | 0.01935 | 0.02849 | 0.00971 |
| Anterior-posterior | 23-25 | Palatal length | 0.036 | 0.054 | 0.353 | 0.00053 | 0.23522 | 0.00176 |
| Anterior-posterior | 24-25 | Posterior palate length | 0.018 | 0.043 | 0.049 | 0.00005 | 0.05895 | 0.00214 |
| Anterior-posterior | 30-34 | Upper mandibular length, left side | 0.087 | 0.179 | 0.382 | 0.02631 | 0.37011 | 0.05026 |
| Anterior-posterior | 31-36 | Upper mandibular length, right side | 0.115 | 0.151 | 0.481 | 0.02656 | 0.45495 | 0.04815 |
| Anterior-posterior | 32-35 | Lower mandibular length, left side | 0.101 | 0.133 | 0.384 | 0.00671 | 0.34336 | 0.02677 |
| Anterior-posterior | 33-37 | Lower mandibular length, right side | 0.099 | 0.181 | 0.100 | 0.00452 | 0.46276 | 0.01534 |
| Transversal | 7-8 | Anterior zygomatic width -facial width | 0.019 | 0.156 | 0.013 | 0.00355 | 0.29791 | 0.02138 |
| Transversal | 9-10 | Inter-orbital width | 0.083 | 0.245 | 0.060 | 0.32860 | 0.28691 | 0.04322 |
| Transversal | 11-12 | Inter-zygomatic arches width | 0.208 | 0.147 | 0.468 | 0.00215 | 0.28731 | 0.01801 |
| Transversal | 13-14 | Zygomatic width - skull width | 0.059 | 0.340 | 0.036 | 0.04530 | 0.25789 | 0.26518 |
| Transversal | 26-27 | Width of ISS | 0.434 | 0.405 | 0.194 | 0.04331 | 0.47779 | 0.02278 |
| Transversal | 28-29 | Width of SOS | 0.228 | 0.323 | 0.106 | 0.35851 | 0.24914 | 0.43030 |
| Transversal | 30-31 | Inter-condile width | 0.144 | 0.247 | 0.149 | 0.02005 | 0.45618 | 0.02779 |
| Transversal | 32-33 | Mandibular width | 0.105 | 0.406 | 0.086 | 0.00278 | 0.47993 | 0.00323 |
| Vertical | 2-24 | Facial height | 0.447 | 0.335 | 0.234 | 0.30619 | 0.35374 | 0.15225 |
| Vertical | 3-21 | Anterior-cranial height | 0.195 | 0.288 | 0.440 | 0.00028 | 0.27884 | 0.00998 |
| Vertical | 4-22 | Mid-cranial height- cranial height | 0.146 | 0.092 | 0.345 | 0.07310 | 0.25384 | 0.08045 |
| Vertical | 5-20 | Posterior-cranial height | 0.058 | 0.042 | 0.128 | 0.02161 | 0.17200 | 0.07144 |
| Vertical | 30-32 | Mandibular height, left side | 0.313 | 0.487 | 0.215 | 0.19290 | 0.26221 | 0.31710 |
| Vertical | 31-33 | Mandibular height, right side | 0.457 | 0.345 | 0.295 | 0.11479 | 0.18538 | 0.04735 |

For more information on Supplementary Material and for details on the different file types accepted, please see [here](https://www.frontiersin.org/guidelines/author-guidelines#supplementary-material).

## Supplementary Figures

##
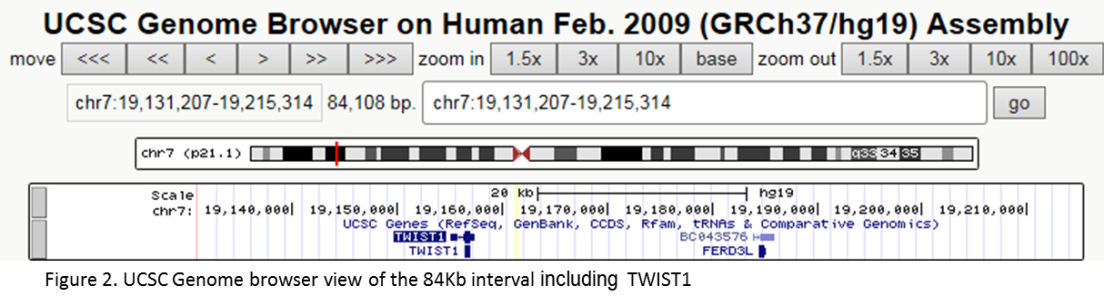
Supplementary Figure 1: UCSC genome browser of 84Kb including *TWIST1* (7p21.1: 19131207-19215314 bps) with an average resolution of a called variant every 667 bps in 272/277 individuals attempted.

**Supplementary Figure 2. PC3, which explains 8% of the variation, depicts changes in ramus height and mandibular body length and is associated with the rare allele rs218900 in *TWIST1.***


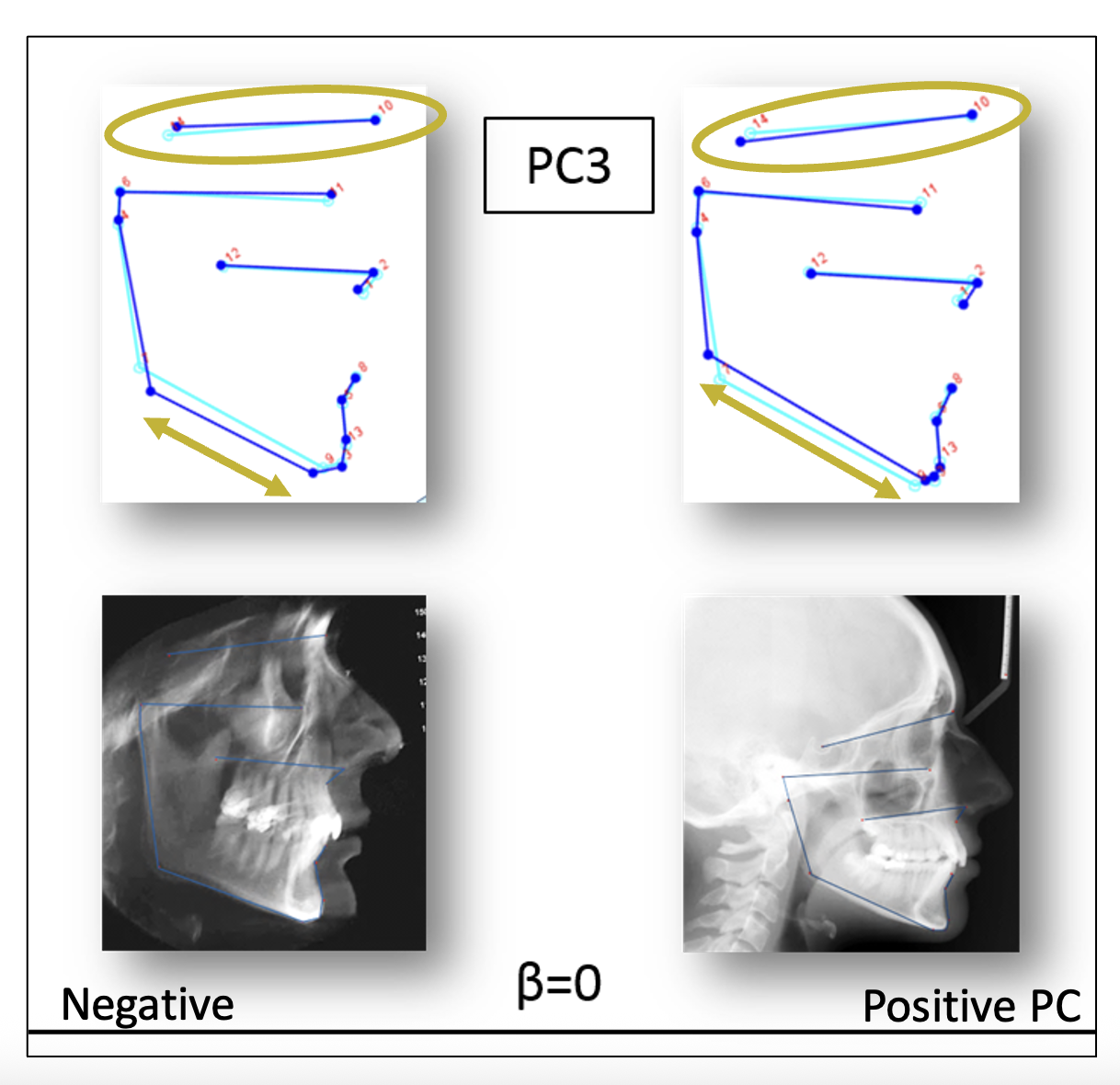

Supplement: Supplementary file 1 [file Supplementaryfile1.docx]
